# High doses of synthetic antioxidants induce premature senescence in cultivated mesenchymal stem cells

**DOI:** 10.1038/s41598-018-37972-y

**Published:** 2019-02-04

**Authors:** Ju. S. Kornienko, I. S. Smirnova, N. A. Pugovkina, Ju. S. Ivanova, M. A. Shilina, T. M. Grinchuk, A. N. Shatrova, N. D. Aksenov, V. V. Zenin, N. N. Nikolsky, O. G. Lyublinskaya

**Affiliations:** Department of Intracellular Signaling and Transport, Institute of Cytology, Russian Academy of Sciences, Tikhoretsky pr. 4, St.Petersburg, 194064 Russia

## Abstract

Stress-induced premature senescence program is known to be activated in cells by various genotoxic stressors, and oxidative stress is considered to be the main of those. To this end, many studies discover antioxidants as protective anti-aging agents. In the current study, we examined the effects of different antioxidants (Tempol, resveratrol, NAC, DPI) on the mesenchymal stem cells maintained in normal physiological conditions. We used high, but non-cytotoxic antioxidant doses which are widely used in laboratory practice to protect cells from oxidative damage. We show that these substances induce reversible block of cell proliferation and do not cause any genotoxic effects when applied to the quiescent cells. However, the same doses of the same substances, when applied to the proliferating cells, can induce irreversible cell cycle arrest, DNA strand breaks accumulation and DNA damage response activation. As a consequence, antioxidant-induced DNA damage results in the stress-induced premature senescence program activation. We conclude that high doses of antioxidants, when applied to the proliferating cells that maintain physiological levels of reactive oxygen species, can cause DNA damage and induce premature senescence which suggests to re-estimate believed unconditional anti-aging antioxidant properties.

## Introduction

Stem cell senescence is considered an important hallmark of aging *in vivo*^1,2^. Recently, it has been found that premature senescence of stem cells is associated with preliminary aging disorders, including Werner syndrome and Hutchinson-Gilford progeria syndrome^3,4^. In contrast to the conditions of aging disorders which are extremely rare *in vivo*, *ex vivo* premature senescence of stem cells is a widely observed event. Activation of premature senescence program has been intensively studied in cultured cells and has been shown to induce proliferation arrest, senescence-like phenotype, as well as global alterations in cell secretome^5^. Premature aging of cultured human stem cells is a serious barrier to the development of tissue engineering and cell therapy technologies for the regenerative medicine applications^6^. Exhausting of cell proliferation impedes cell propagation which is required for providing a source of transplantable cells. Besides, senescent cells, when injected into an organism for the therapeutic needs, can induce inflammation and oncological transformation of healthy tissues due to the potentially harmful secretory phenotype^7^.

Premature aging of cultured stem cells is usually associated with the exposure of cells to the environmental stress factors^8,9^. The concept of stress-induced premature senescence (SIPS) was first introduced in 2000 by Dr. Olivier Toussaint and co-workers^10,11^. Sublethal oxidative stress was shown to arrest proliferation and promote accumulation of senescence-associated molecular hallmarks (increased activity of cyclin-dependent kinase inhibitor p21^Waf1/Cip1^ (p21) and β-galactosidase (SA-β-gal), as well as lack of phosphorylated retinoblastoma gene product (ppRb)) in diploid fibroblasts^12^. Later on, it was proven that along with fibroblasts, many other normal human cells (including stem cells) are susceptible to SIPS program activation^2,5,9,13^. Various genotoxic agents, such as radiation^14^, cytostatic agents^15,16^, heat shock^17,18^ etc. are well-established inducers of SIPS. However, oxidative stress is believed to be the major cause of SIPS program activation in normal cells^8,19,20^. Enhanced production of reactive oxygen species often accompanies stress conditions induced by various environmental factors (UV radiation, X-ray exposure, toxicants) and SIPS, in this case, may appear not only as a direct consequence but also as a side effect of these harmful impacts^21^.

Since oxidative stress is a well-known inducer of premature senescence, a lot of research showing beneficial effects of antioxidants (AOs) has been performed both *in vitro* and *in vivo*^22,23^. In most of these studies, protective and anti-aging antioxidant (AO) effects occurred in the case of induced oxidative stress conditions. However, the full range of effects produced by AO substances on cells maintained in normal physiological conditions still needs to be characterized. Over the last years, works showing that AOs are able to act as stressors themselves when applied to normal cells under non-oxidative conditions started to appear^24–33^. Although in some of these studies AOs were shown to induce proliferation arrest^24–31^, DNA damage^32,33^, chromosomal abnormalities^32^, and apoptosis induction^30,33^, to date, there have been no discussions on potential pro-aging AO properties. Here, we prove that sublethal doses of different synthetic AOs can activate the SIPS program in proliferating mesenchymal stem cells as a consequence of DNA damage induction. Thus, we conclude that anti-aging properties are highly dependent on the applied doses of AOs.

In our previous work^34^ we showed that reactive oxygen species (ROS) are essential for human mesenchymal stem cells originating from different organs to exit quiescence state and that AO treatments can block initiation of the proliferating cycle. Within the current study, we aimed to dissect whether different AOs (see Table 1) affect the proliferation of awakened cycling adult stem cells. To this end, we exploited endometrial human mesenchymal stem cells (eMSCs)^35^ as a model of normal and actively proliferating in culture adult human stem cells. AOs employed within this study (Tempol^36^, resveratrol^37^, N-acetyl-L-cysteine (NAC)^38^, Diphenyleneiodonium (DPI^39^) have different mechanisms of action (see Table 1). All of them, being either of natural or synthetic origin, are known to have a broad range of nonspecific effects on cells. Our aim was to specify the effects that were common for all examined AOs applied at high but non-lethal concentrations.

**Table 1:** List of the antioxidants used within the study.

Antioxidant	Origin	Antioxidant activity	Final concentrations
Tempol (4-Hydroxy-TEMPO)	Synthetic nitroxide	Low molecular weight redox cycling nitroxide, superoxide dismutase mimetic, free radical scavenger^36^	1–2 mM
Resveratrol (3,4′,5-trihydroxystilbene)	Natural polyphenolic phytoalexin	Free radical scavenger and antioxidant enzymes activity promoter^37^	10–60 *μ*M
N-acetyl-L-cysteine (NAC)	Synthetic acetylated cysteine residue	Aminothiol, a source of sulfhydryl groups to cells as a precursor of intracellular cysteine and reduced glutathione (GSH), free radical scavenger^38^	10–40 mM
Diphenyleneiodonium (DPI)	Small iodine- containing bioactive molecule	Uncompetitive non-specific inhibitor of flavoenzymes (particularly NADPH oxidase)^39^	1–2 *μ*M

## Results

### Antioxidants diminish intracellular ROS

**Figure 1:**
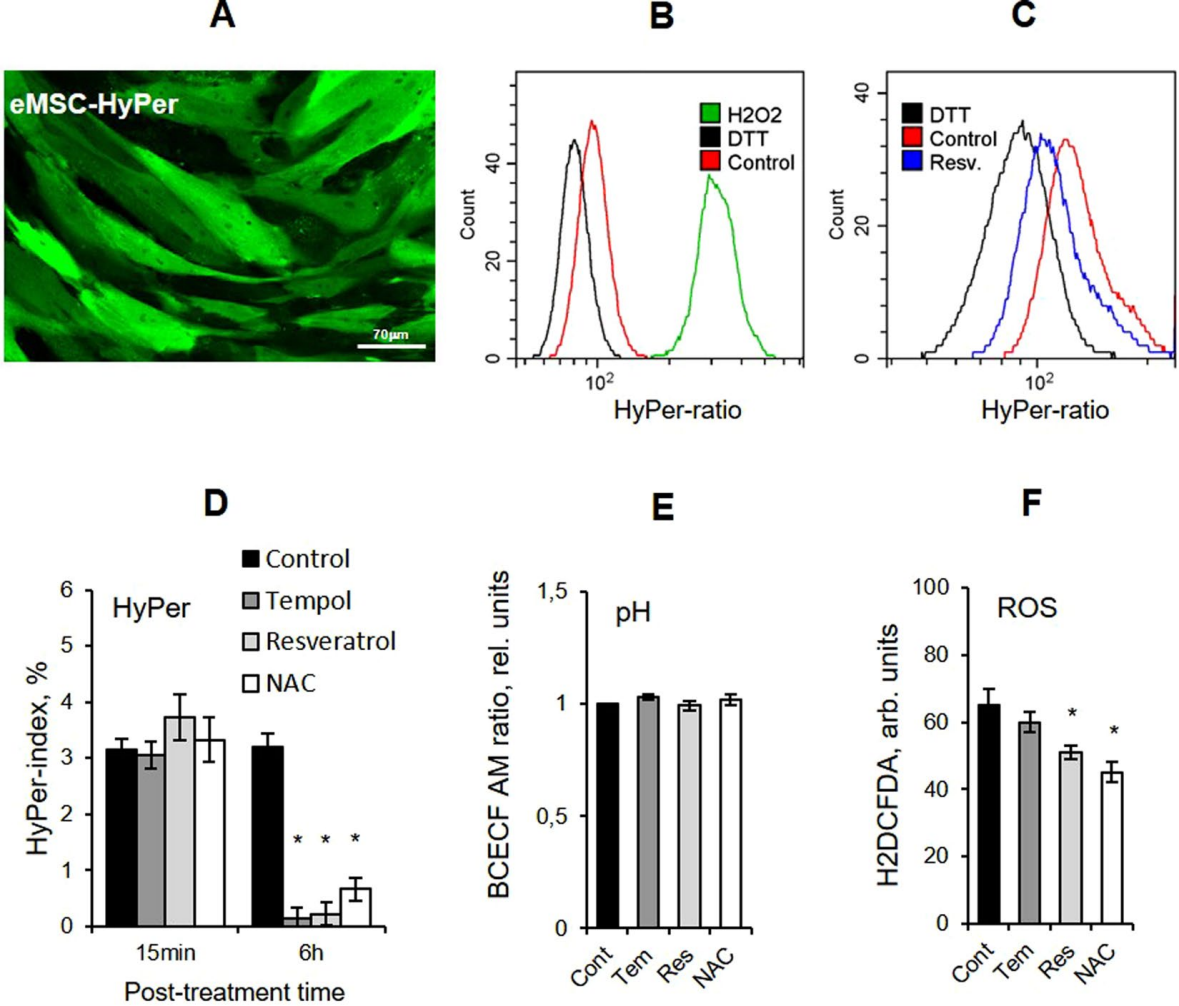
Antioxidant treatments cause a decrease of the ROS level in cells. (**A**) Confocal microscopy image of the eMSC-HyPer cells (scale bar = 30 μM). (**B**) Flow cytometry ratiometric histograms of control eMSC-HyPer cells, as well as cells treated with H_2_O_2_ (1 mM, 5 min) and DTT (30 mM, 10 min). (**C**) Flow cytometry ratiometric histograms of the control eMSC-HyPer cells and cells treated with resveratrol (40 μM, 2 h) and DTT (30 mM, 10 min) reveal decrease of the HyPer-ratio after resveratrol treatment. (**D**) HyPer-index, estimated for the control and AO-treated eMSC-HyPer cells after 15-min and 6-hour incubation with AOs, indicates a decrease in the basal H_2_O_2_ concentration after 6-hour incubation. (**E**) BCECF AM ratio, measured by flow cytometry in the control and AO-treated eMSCs stained with BCECF AM dye, confirms that 6-hour incubation with AOs does not affect cellular pH (signal ratio was normalized to the control value). (**F**) Flow cytometry analysis of the mean fluorescence in the eMSCs incubated with AOs for 6 hours and then stained with H_2_DCFDA, ROS-sensitive dye. Measurements do not reveal the pro-oxidatve effects of AOs after 6-hour incubation. All results are presented as mean ± SD of three measurements, *p < 0.05, ANOVA test. Abbreviations: ROS, reactive oxygen species; DTT, dithiothreitol; H_2_O_2_, hydrogen peroxide; eMSCs, endometrial mesenchymal stem cells; eMSC-HyPer cells, eMSCs stably expressing HyPer-cyto protein; AOs, antioxidants, namely Tempol (Tem, 2 mM), resveratrol (Res, 40 μM), N-acetyl-L- cysteine (NAC, 20 mM); HyPer-ratio, ratio of EX488/FL525 and EX405/FL525 signals multiplied by 100; BCECF AM, pH-sensitive ratiometric dye; BCECF AM ratio, ratio of EX488/FL525 and EX405/FL525 signals multiplied by 100; HyPer-index, quantification of HyPer oxidation calculated as follows: HyPer-index = (Rcells − R_DTT_/(R_H2O2_ − R_DTT_), where Rcells – HyPer-ratio in the cells of interest, R_DTT_ and R_H2O2_ – HyPer-ratio in the control cells treated with DTT (30 mM, 10 min) and H_2_O_2_ (1 mM, 5 min) respectively; H_2_DCFDA, 2′,7′-dichlorodihydrofluorescein diacetate; SD, standard deviation.

To start with, we defined a cytotoxic threshold for each AO substance employed within the study and used AO doses which were well below these thresholds in the following experiments (Supplementary Fig. S1A). Then we confirmed that all AOs applied within the chosen concentration range inhibited intracellular ROS and did not cause instantaneous pro-oxidative effects (Fig. 1). We generated stably expressing HyPer eMSC line (eMSC-HyPer) to monitor changes in the intracellular ROS level in response to AO treatments (Fig. 1A). HyPer is a genetically encoded fluorescent probe for hydrogen peroxide (H_2_O_2_), which contains the H_2_O_2_-sensitive regulatory domain of the *E*. *coli* transcription factor OxyR and circularly permuted yellow fluorescent protein (cpYFP) integrated into the sequence of OxyR^40^. HyPer is a highly sensitive ratiometric probe for H_2_O_2_ detection in living cells and can be targeted to various cell compartments^41–44^. In this study, we exploited the ratiometric flow cytometry analysis of cells expressing HyPer in cell cytoplasm^45^. By using two-laser flow cytometer, we directly analyzed ratio of EX488/FL525 and EX405/FL525 signals (further referred to as a HyPer-ratio) (Fig. 1B). It appeared that HyPer-ratio of eMSC-HyPer cells clearly decreased after AO treatments. Total reduction and total oxidation of HyPer with 30 mM dithiothreitol (DTT) and 1 mM H_2_O_2_ respectively (Fig. 1B) were exploited for the quantification of HyPer oxidation range^42^. We defined the shift of HyPer-ratio from the totally reduced state (considered as 0%) towards totally oxidized state (considered as 100%) as a HyPer oxidation index quantified in %^45^ and estimated these indexes in both control cells and cells treated with AOs for 15 minutes and 6 hours. While short incubations did not affect HyPer-index, 6-hour treatments resulted in attenuated HyPer oxidation in proliferating cells (Fig. 1D) which proved that employed AO treatments did not cause pro-oxidative effects in eMSC-HyPer cells. Since HyPer is a pH-sensitive probe^41^, intracellular pH changes in response to AO treatments were monitored in parallel experiments with the use of fluorescent dye 2′,7′-bis-(2-carboxyethyl)-5-(and-6)-carboxyfluorescein, acetoxymethyl ester (BCECF AM). 6-hour AO treatments had no noticeable effect on the acidity in cells (Fig. 1E).

Moreover, we checked the overall ROS level in proliferating cells treated with AOs for 6 hours. To this end, eMSCs were stained with a fluorescein-containing cell-permeable probe 2′, 7′- dichlorodihydrofluorescein diacetate (H_2_DCFDA). H_2_DCFDA becomes fluorescent in the result of the multi-step intracellular oxidation process and is routinely used as a general indicator of the cellular redox-status^46,47^. Fluorescence signal measured with flow cytometer in the cells treated with the aforementioned AOs, excluding Tempol, was lower than that in the control cells (Fig. 1F). Tempol was the only exception to the rule: the difference between the fluorescence signals in control and Tempol-treated cells was estimated to be insignificant. In addition, ROS level measured with the use of H_2_DCFDA dye was being lower than that in control cells within at least 20 hours after AO application. The data for resveratrol is presented as an example (Fig. S1B).

### Antioxidants disrupt cell proliferation when applied to both quiescent and proliferating cells

To study on how AOs affect cells with different proliferation status, we used cell cycle synchronization approach. Cells were synchronized in the G_0_ phase of the cell cycle by serum starvation and then stimulated for proliferation by serum supplementation. AOs were added to the cell medium either two hours after serum stimulation, when the cells were primarily in G_0_/G_1_-phase of the cell cycle (experimental design further referred to as “Quiescence cells antioxidant treatment”, “Q-AO-treatment”), or 14 hours later, in the early S-phase of the cell cycle (scheme further referred to as “Proliferating cells antioxidant treatment”, “P-AO-treatment”).

**Figure 2:**
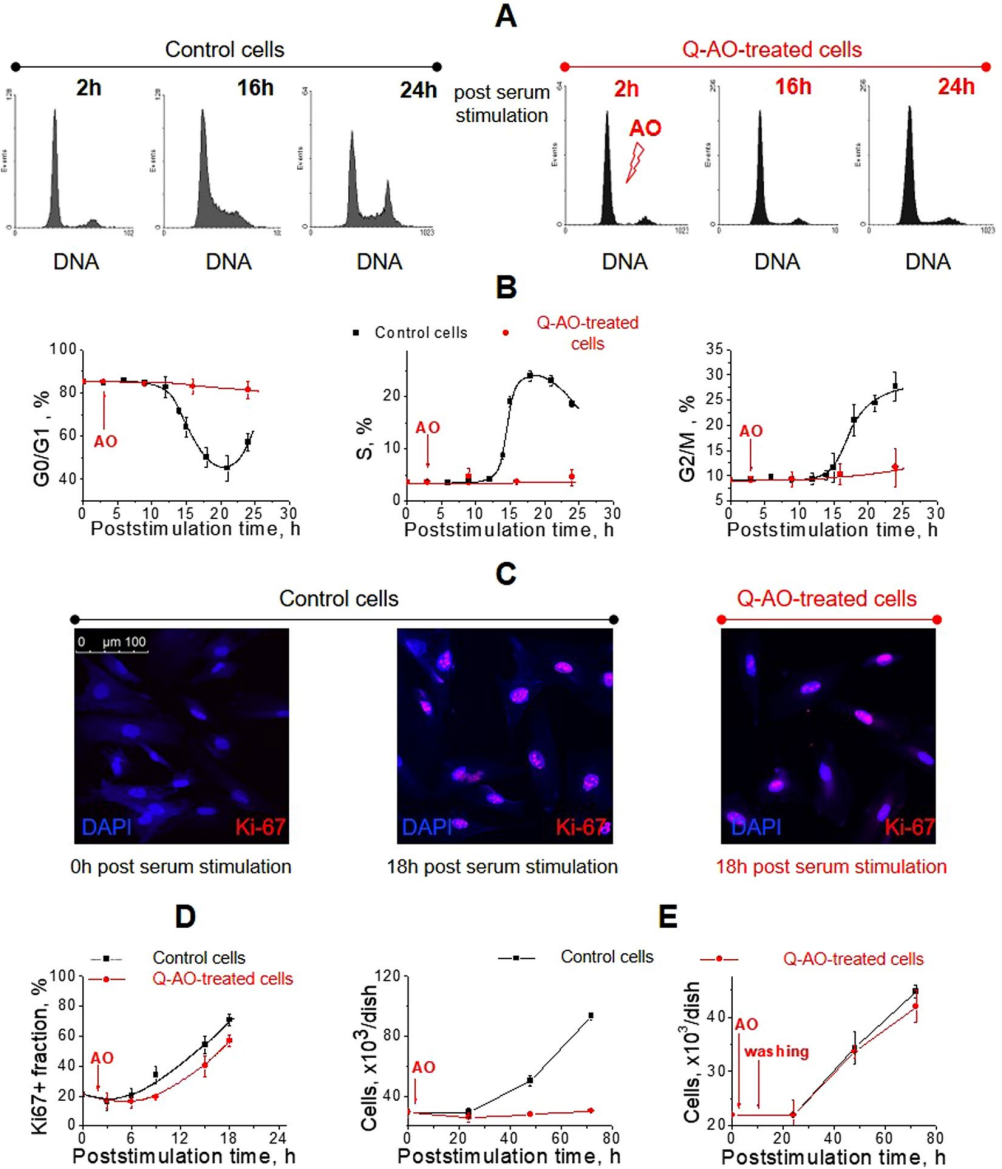
Cells treated with antioxidants at quiescent state are reversibly arrested in the late G_1_ phase. (**A**,**B**) Cell cycle progression of the synchronized eMSCs after serum stimulation. Flow cytometry histograms (**A**) and quantification of G_0_/G_1_, S, and G_2_/M cell fractions (**B**) in the control and Q-AO-treated eMSCs reveal G_0_/G_1_-block of cell proliferation induced by AO treatment. Data are presented as mean ± SD of three independent experiments. (**C**,**D**) Immunofluorescence analysis of Ki-67 (proliferation marker protein) expression in the control and Q-AO-treated eMSCs. Representative images (**C**) and dynamics of Ki-67 + cell fraction accumulation (**D**) confirm not G_0_, but G_1_-blocking of cell proliferation after Q-AO-treatment. At least 7 images with at least 30 cells were processed for each time point. Data are presented as mean ± SD. (**E**) Cell growth curves for Q-AO-treated eMSCs, either constantly incubated with AO (left panel) or washed of AO after 8-hour incubation (right panel), show the reversible character of AO-induced G_1_-block (n = 3). Results are the mean ± SD of three measurements. Abbreviations: eMSCs, endometrial mesenchymal stem cells; AO, antioxidant Tempol at 1 mM concentration; Q-AO-treated cells, eMSCs exposed to 1 mM of Tempol at 2 h post-serum stimulation; DAPI, 4′,6-diamidino-2-phenylindole; SD, standard deviation.

In the first batch of experiments, cell proliferation was monitored in quiescent eMSC cultures treated with AOs soon after stimulation of proliferation (see Figs 2 and S2). Within 24 hours after serum stimulation, control cells exited quiescence and progressed through G_1_, S and G_2_/M phases of the proliferation cycle, whereas cells exposed to AOs were arrested in G_0_/G_1_ phase (See Fig. 2A,B for the Tempol treatment; See Fig. S2D for treatment with the other AOs), in consistency with the previously published studies^34^. Cell cycle block had dose-dependent character (Fig. S2A,B). Along with the cell cycle phase distribution, we monitored expression of the Ki-67 protein (proliferation marker which is being expressed during G_1_, S and G_2_/M phases of the cell cycle but is completely lacked in G_0_ phase^48^). It appeared that Ki-67 accumulation dynamics in AO-exposed cells was only slightly slower than in control eMSCs (Figs 2C,D and S2C) which evidenced that AO-treated cells were blocked not in G_0_ but in G_1_ phase of the cycle. Interestingly, cells were able to restore their proliferation capacity after removal of AO. Treated with Tempol for 8 hours and subsequently washed with the fresh growth medium eMSCs exhibited the same proliferation rate as the untreated cells (Fig. 2E).

**Figure 3:**
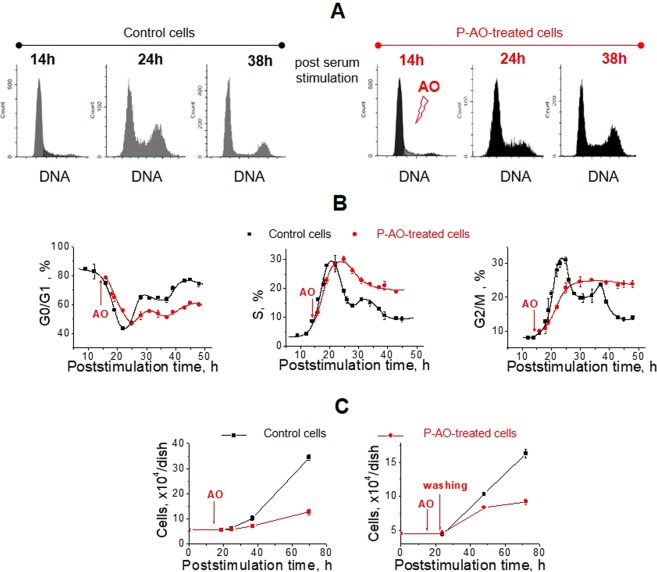
Proliferating cells treated with antioxidants progress through the S phase slowly and are eventually accumulated in the G_2_/M phase. (**A**,**B**) Cell cycle progression of the synchronized eMSCs after serum stimulation. Representative flow cytometry histograms (**A**) and quantification of G_0_/G_1_, S, and G_2_/M cell fractions (**B**) in P-AO-treated eMSCs reveal slowdown of the S phase progression and further G_2_/M-block of cell proliferation. The data shown are the average of four independent experiments ± SD. (**C**) Cell growth curves for P-AO-treated eMSCs, either constantly incubated with AO (left panel) or washed of AO after 8-hour incubation (right panel), confirm the irreversible character of proliferation block induced by P-AO-treatment. Average of three measurements ± SD. Abbreviations: eMSCs, endometrial mesenchymal stem cells; AO, antioxidant Tempol at 1 mM concentration; P-AO-treated cells, eMSCs exposed to 1 mM of Tempol at 14 h post-serum stimulation; SD, standard deviation.

In the second batch of experiments, we checked whether the same doses of AOs can inhibit self-renewal of the proliferating eMSC cultures (see Figs 3 and S3). Cell cycle analysis showed that in the case of P-AO-treatment, Tempol exposure caused a dose-dependent slowdown of DNA synthesis phase and eventually resulted in G_2_/M block of cell proliferation (Fig. 3A,B). In contrast to Q-AO-treatment, after incubation with Tempol for 8 hours and subsequent cell washing, P-AO-treated eMSCs were not able to recover their proliferation capacity (Fig. 3C) which indirectly indicated potentially damaging effects of AOs. Exposure of eMSCs to the other AOs produced effects similar to those of Tempol: treatment of proliferating eMSCs with sub-cytotoxic doses of AOs decelerated DNA synthesis phase progression and then blocked cells in G_2_/M-phase (see Fig. S3 in the Supplement).

### Antioxidants can cause DNA damage when applied to proliferating cells

**Figure 4:**
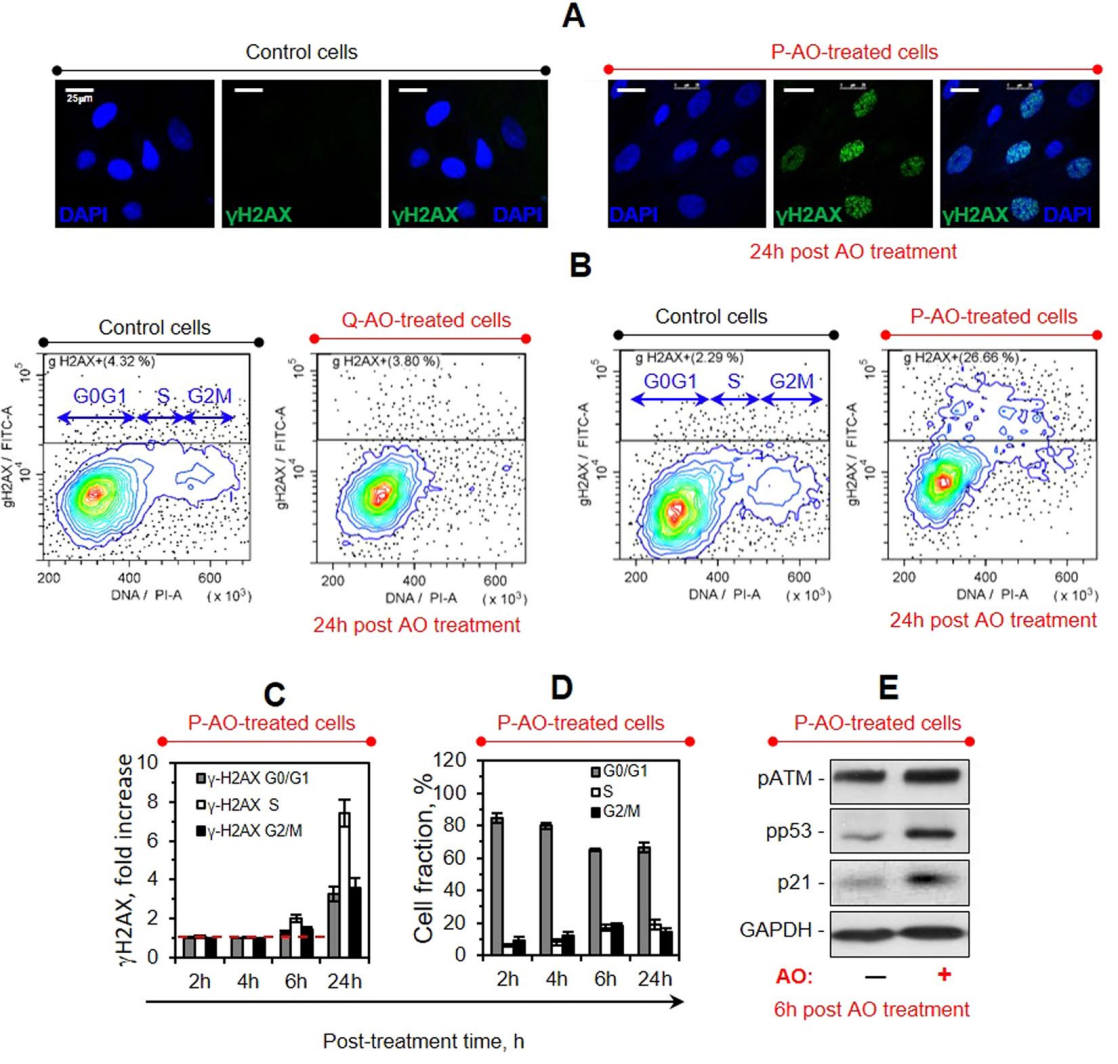
Treatments with antioxidants induce replicative stress in P-AO-treated cells. (**A**) Immunofluorescence analysis of the P-AO-treated eMSCs reveals γH2AX foci accumulation within 24 h post AO treatment, scale bar = 25 μm. (**B**–**D**) Flow cytometry studies of the γH2AX + cell distribution among the cell cycle phases in the control, P-AO-treated and Q-AO-treated cells performed at different time points after the cell treatments. Representative dotplots of cell cycle distributions of the eMSCs stained with γH2AX antibody (**B**), mean γH2AX immunofluorescence signals in G_0_/G_1_, S and G_2_/M cell fractions normalized to the control values (**C**) and corresponding cell cycle distributions (**D**) are shown. The data show slow accumulation of γH2AX foci in cells replicating their DNA, which is correlated with the decelerated S phase progression in P-AO-treated eMSCs. Mean values ± SD of three independent experiments are shown. (**E**) Western blot analysis of the DDR-related protein (pATM, pp53, and p21) expression in P-AO-treated eMSCs after 6-hour AO treatment. The blot was stained with Ponceau S Red and then cut at the appropriate molecular weights of proteins of interest. Abbreviations: eMSCs, endometrial mesenchymal stem cells; γH2AX phosphorylated histone H2AX; pATM, phosphorylated ATM kinase; pp53, phosphorylated p53; p21, cyclin-dependent kinase inhibitor p21^waf1/cip1^; AO, antioxidant Tempol at 2 mM concentration; P-AO-treated cells, eMSCs exposed to 2 mM of Tempol at 14 h post-serum stimulation; DDR, DNA damage response; SD, standard deviation.

Since the irreversibility of self-renewal block is often associated with DNA damage response, we next examined DNA integrity in AO-exposed eMSCs. Our studies (Figs 4As S4C–F) revealed the accumulation of phosphorylated histone H2AX (γH2AX) in the proliferating eMSCs after 24-hour exposure to AOs, which is a well-known hallmark of DNA strand breaks formation^49^. Flow cytometry analyses (Figs 4Bs S4E) detected DNA strand breaks only in proliferating eMSCs, but not in the quiescent eMSCs after their 24-hours exposure to AOs. Analysis of γH2AX accumulation dynamics and its comparison to the cell cycle distribution dynamics after treatment of proliferating eMSCs with Tempol showed that the fraction of cells with DNA strand breaks increased with time in accord with the accumulation of cells blocked in the S-phase (Fig. 4C,D). Slow dynamics of DNA breaks accumulation together with the decelerated S phase progression indicated that AOs disturbed DNA replication process in proliferating cells. Whereas resveratrol, DPI and Tempol were equally effective in DNA damage induction, NAC was less effective: its sub-cytotoxic concentrations induced slow accumulation of γH2AX foci, while higher concentrations induced rounding and detachment of cells (data not shown).

To determine whether the induction of DNA breaks caused a DNA damage response (DDR) activation^50^, we assessed molecular markers of the DDR such as phosphorylation of ATM (аtaxia telangiectasia mutated) kinase and its downstream target p53 transcription factor, as well as accumulation of p21 protein. Western blot analysis (Figs 4E and S4G), as well as immunofluorescence studies (Fig. S4A,B), showed that nuclear γH2AX foci formation was accompanied with activation of the ATM/p53/p21 pathway. This observation was true again only for proliferating, but not quiescent cells treated with AOs (Fig. S4H). Based on these results, we conclude that high doses of AOs can cause DNA damage and induce DNA damage response activation when applied to the proliferating eMSC cultures.

### Antioxidants induce activation of stress-induced premature senescence program in proliferating cells

**Figure 5:**
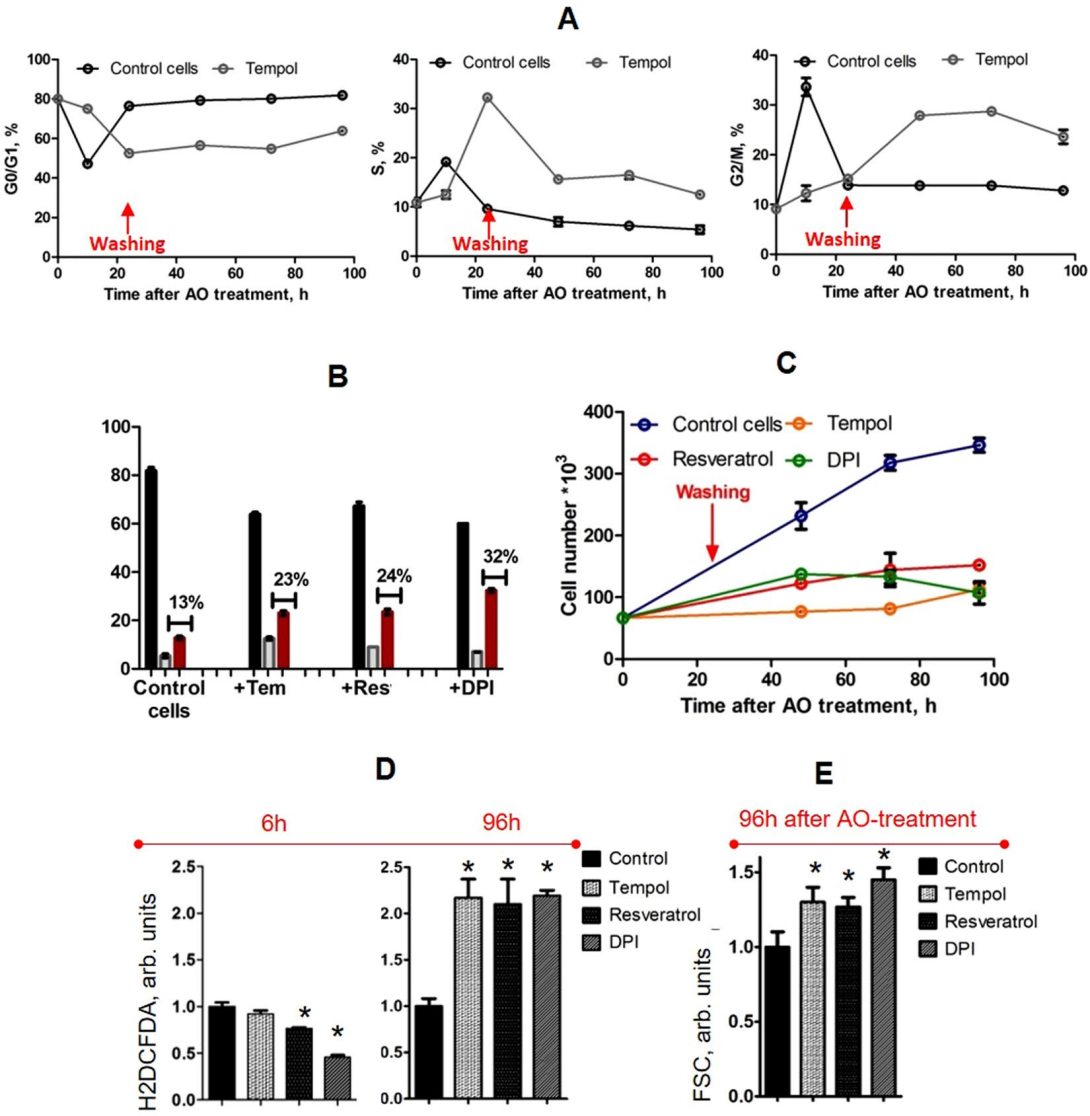
P-AO-treated cells irreversibly stop to self-renew, enlarge and accumulate ROS. (**A**) Cell cycle progression of the synchronized eMSCs after 24h-P-AO treatment (with Tempol) and subsequent washing. Quantification of the G_0_/G_1_, S and G_2_/M cell fractions in P-AO-treated eMSCs reveals S-G_2_/M-block of cell proliferation. (**B**) Cell cycle distributions of the control and P-AO-treated eMSCs after 24-hour treatment, washing and three day cultivation (96 hours after treatment or 72 hours after washing): G_2_/M-blocking after Tempol, resveratrol, NAC and DPI applications (flow cytometry studies). (**C**) Growth curves after 24h-P-AO-treatment with Tempol, resveratrol or DPI and subsequent washing show irreversibility of cell proliferation block. (**D**) Flow cytometry analysis of the mean cell fluorescence in the P-AO-treated cells incubated with AOs for 6 hours (left panel) or P-AO-treated cells incubated with AOs for 24 hours, washed and cultured for three more days (96 hours after AO treatment or 72 hours after washing, right panel) and then stained with H_2_DCFDA, ROS- sensitive dye. Measurements do not reveal the pro-oxidatve effect of AOs after 6-hour incubation, however, they show a significant increase in ROS level 4 days post AO treatment. (**E**) Flow cytometry estimation of geometric cell sizes by the means of forward scattering signal (FSC) in the P-AO-treated cells incubated with AOs for 24 hours, washed and cultured for three more days (96 hours after AO treatment or 72 hours after washing, right panel). Results illustrate significant enlargement of the cells 4 days after AO treatment. All data are shown as an average of at least three independent experiments ± SD. *p < 0.05, ANOVA test. Abbreviations: ROS, reactive oxygen species; eMSCs, endometrial mesenchymal stem cells; AOs, antioxidants, namely Tempol (Tem, 2 mM), resveratrol (Res, 60 μM), Diphenyleneiodonium (DPI, 2 μM); P-AO-treated cells, eMSCs exposed to antioxidants at 14 h post-serum stimulation; SD, standard deviation.

We next examined the fate of proliferating cells, treated with AOs for 24 hours and then washed with a fresh medium. During 3 days after AO washing, we monitored the cell cycle phase distribution, dynamics of cell growth and intracellular ROS level (see Figs 5 and S5). As it turned out, AOs, applied to the proliferating eMSCs for 24 hours, caused an irreversible accumulation of cells in G_2_/M-phases of the cell cycle (Figs 5A,B and S5A,B). Even though cell growth was arrested (Fig. 5C), AO treatments did not result in any detectable cell death induction (Fig. S5C). Interestingly, whereas the ROS level measured with H_2_DCFDA in AO-treated cells was maintained at a lower level than that in control cells within 24 hours of incubation with AOs (see Figs 5D and S1B), after cell washing we observed the delayed increase in ROS (Fig. 5D). As the latter was observed several days after removal of AOs, we consider this effect to be a consequence of genotoxic stress rather than a direct pro-oxidative effect of applied AOs. Besides, elevation of ROS and prolonged inhibition of self-renewal were accompanied with the cell enlargement assessed by the forward scattering signal (Fig. 5E), that usually accompany SIPS program activation in cells in response to DNA damage^9^. Therefore, these results indirectly indicate that genotoxic stress, induced in proliferating eMSCs by AOs, may result in premature senescence program induction rather than cell death.

**Figure 6:**
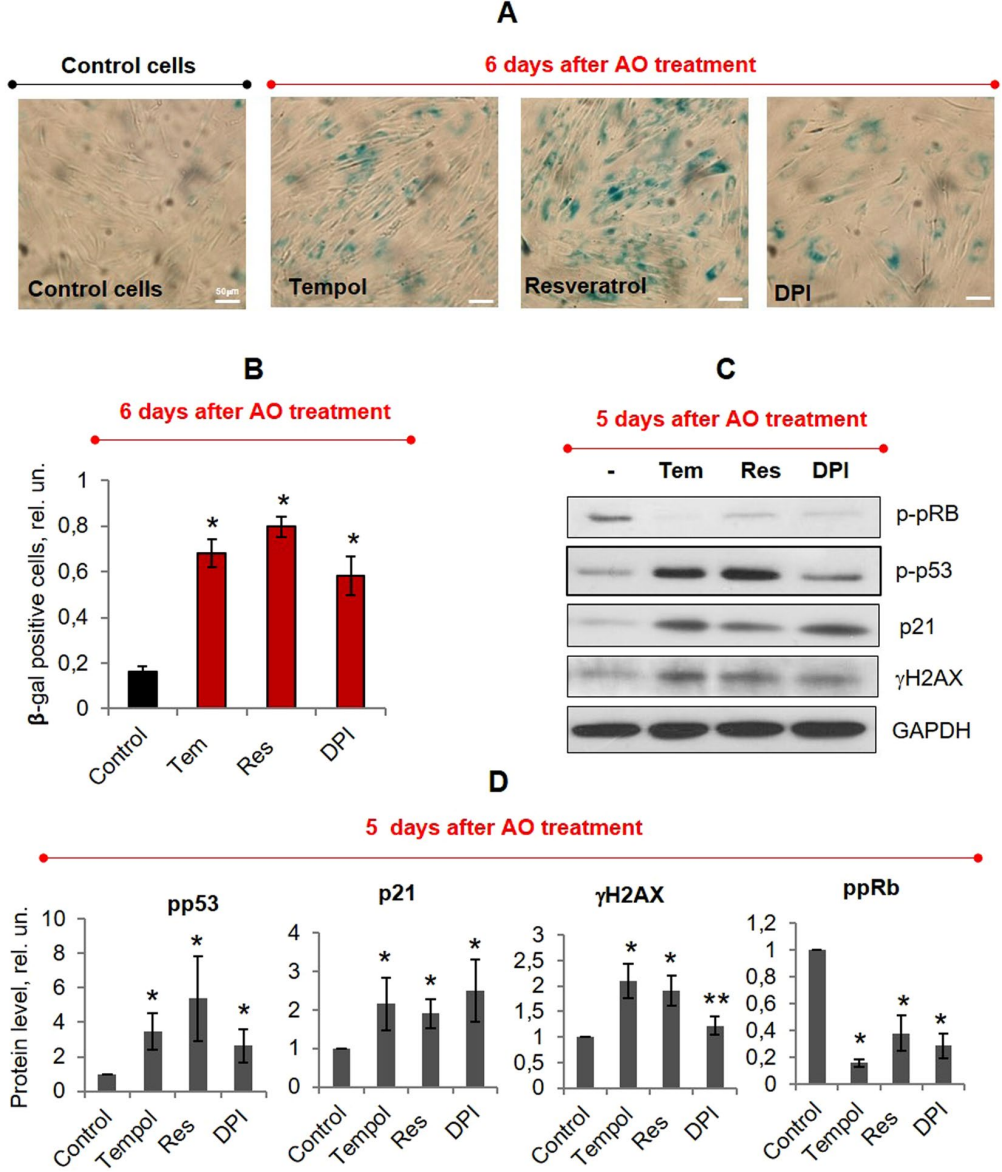
P-AO-treated cells possess all hallmarks of senescent cells. (**A**) SA-β-Gal staining 6 days after 24h-P-AO-treatment (or 5 days after washing, 2 days after the last splitting) with Tempol (Tem, 2 mM), resveratrol (Res, 60 μM), DPI (2 μM). Senescent cells were detected with SA-β-Gal staining kit, Ob: 20x, scale bar = 50 μm. (**B**) Quantification of SA-β-Gal positive cells. At least 500 cells from the different fields of view were analyzed with the use of ImageJ software. (**C**) Western blot analysis of the SIPS-related protein expression (p-pRb, p-p53, p21, γH2AX) in P-AO-treated eMSCs 5 days after 24h-P-AO-treatment (or 4 days after washing, 1 day after the last splitting). The blot was stained with Ponceau S Red and then cut at the appropriate molecular weights of proteins of interest. (**D**) Quantification of protein bands. The samples were derived from three independent experiments and western blots were run in parallel. All data are shown as the mean of at least three independent experiments ± SD. *p < 0.05, **p < 0.10, ANOVA test. Abbreviations: eMSCs, endometrial mesenchymal stem cells; AO, antioxidant, namely Tempol (Tem, 2 mM); resveratrol (Res, 60 μM); diphenyleneiodonium (DPI, 2 μM); P-AO-treated cells, eMSCs exposed to antioxidants at 14 h post-serum stimulation; γH2AX, phosphorylated histone H2AX; p-p53, phosphorylated p53; p21, cyclin-dependent kinase inhibitor p21^waf1/cip1^; p-pRb, phosphorylated retinoblastoma protein; SA-β-Gal, senescence-associated β-galactosidase, SIPS, stress-induced premature senescence; SD, standard deviation.

To examine this, we studied the accumulation of molecular markers of stress-induced premature senescence (SIPS) in cells treated with AOs for 24 hours. After cell washing, eMSCs were cultivated for three days and then split to the equal density. Analysis of the SIPS markers accumulation was performed 1–2 days after splitting. We found that even after such long cultivation of cells without any stressors, cells sustained high level of γH2AX protein as well as pp53 (phosphor-p53) and p21 proteins (see Fig. 6C,D). The last indicated inability of the cells to cope with DNA damage caused by P-AO treatment and showed that prolonged proliferation arrest is mediated by p21 expression. Moreover, we detected the high activity of senescence-associated β-galactosidase (SA-β-galactosidase) in P-AO treated cells (see Fig. 6A,B) and lack of phosphorylated pRb protein which are known to be well-established hallmarks of senescence program activation^5,19^.

These data prove that sub-cytotoxic doses of AOs, applied to proliferating eMSCs, cause activation of the SIPS program marked by high levels of p21 and SA-β-galactosidase as well as by the absence of ppRb protein.

## Discussion

Oxidative stress and process of aging are closely related to each other. It has been proven that both acute and chronic oxidative stress lead to induction of the premature senescence program both *in vitro* and *in vivo*^8,9^. Which is why, quite a long time ago, research on anti-aging properties of AOs had been initiated and attracted a lot of public’s attention^51^. First results seemed promising and nowadays for a large number of people the words “antioxidants” and “anti-aging” are related to each other as close, as the words “oxidants” and “aging” are. However, within the last few years, paradigms have been being changed. The works, showing negative effects of AOs on normal, healthy cells which sustain physiological ROS levels, have started to accumulate^24–33^. High doses of AOs of different origin have been shown to disturb regulation of the cell cycle not only in normal but even in cancer cells, therefore, inhibiting their self-renewal^24–31^. This effect may sound beneficial in application to the inhibition of cancer cell proliferation and a lot of research has been done to this end as well^52^. However, in the absence of any pathology, the disturbance of healthy cell self-renewal cannot be considered beneficial. Moreover, in some studies AOs applied at high doses have been shown to induce DNA damage and chromosomal abnormalities in several types of stem cells^32,33^. The last may potentially lead to either apoptosis, premature senescence or oncogenic transformation of stem cells, which is why it seems important to reevaluate the expected benefits of high-dose AO supplementations.

In the current study, we addressed the question of how different AOs affect both quiescent and proliferating mesenchymal stem cells. To this end, we used cell cycle synchronization approach to collect cells in G_0_-phase of the cell cycle and exposed cells to AOs either soon after proliferation stimulation (we called it Q-AO-treatment) or 14 hours after proliferation stimulation (we called it P-AO-treatment). AOs were applied at doses which are widely used for protection of cells under oxidative conditions *in vitro* and *in vivo*^36–38^ and, in the case of resveratrol, even in clinical trials^53^. We showed that Q-AO-treatment caused G_1_-cell cycle arrest which was, however, reversible after AO washing due to the absence of DNA-damaging effects. In contrast, P-AO-treatment blocked cells in S-G_2_/M-phases of the cell cycle irreversibly due to the accumulation of DNA strand breaks and the DNA damage response activation (pATM/pp53/p21 pathway). As a consequence, proliferating mesenchymal stem cells responded to AO-treatments with activation of the SIPS program which manifests itself in the accumulation of well-known hallmarks (an increase of the ROS level, cell enlargement, proliferation arrest, SA-β-galactosidase activity, lack of phosphorylated pRb, an activity of cyclin-dependent kinase inhibitor p21).

Even though elucidation of the concrete causes of AO-induced DNA damage and SIPS is beyond the scope of this study, it is worth noting that three of the four AOs of different origins and mechanisms of action used in experiments were able to induce DNA damage in proliferating, but not quiescent eMSCs. Moreover, accumulation of DNA strand breaks in cells replicating their DNA was not accompanied by the pro-oxidative effects of AOs which are sometimes observed in the case of high-dose AO applications^37^. Having said all this and taking into account previously published works^24–29^, we assume that one of the possible causes of AO-induced damage could be an interference into the cell cycle regulation process. It has been shown that different AOs can induce ubiquitin-dependent degradation of the key S-phase regulating proteins through the activation of APC^Cdh1^ ubiquitin-ligase complex (anaphase promoting complex)^25^. Since some of the AO-affected proteins (such as cyclin A2 and other targets of APC^Cdh1^ complex) are required for the accurate replication, we tend to think that AO-induced degradation of these proteins can disturb replication process, resulting in the replication stress induction. Moreover, in other work^32^ it has been shown that high doses of various AOs can disrupt DNA reparation process, therefore, leading to the inability of cells to repair DNA breaks which occur normally during the DNA replication process. Thereby we tend to speculate that AO-induced DNA damage might appear due to the disturbance of DNA synthesis and/or DNA repair processes and that AOs when applied to the normal stem cells, which sustain physiological ROS level, can act as damaging and pro-aging stressor agents.

Recently, the term “antioxidative stress” has been introduced to describe the disruption of normal cell/tissue functions caused by an overabundance of AOs in an organism^54,55^. However, the number of experimental studies on this topic is quite limited. Among them, studies on the impact of dietary AOs^56^ show that antioxidative stress disturbs metabolic cues, regulating immune response of an organism and therefore promotes progression of various human diseases such as asthma, allergy, and obesity. Besides, it has been established that chronic antioxidative stress resulted from the constant overexpression of AO enzymes^57^, as well as supplementation with some pharmacological AOs, such as N-acetyl-L-cysteine (NAC)^58^, induces the metabolic imbalance referred to as a “reductive stress”. The latter is defined as an abnormal increase in the level of reducing equivalents in the forms of NADH, NADPH, and GSH in the cell. Reductive stress has been shown to disturb cellular respiration metabolism and results in an increased mitochondrial oxidation and cytotoxicity^57–59^. Furthermore, evidence has been provided that reductive stress precedes the development of some pathologies which are usually associated with elevated levels of ROS (cardiomyopathy, chronic hyperglycemia, neurodegeneration)^60–62^. However, by now, no works showing that antioxidative and/or reductive stresses can cause activation of the SIPS program exist. Here we link SIPS and antioxidative/reductive stresses for the first time and we believe that more research on the concrete mechanisms of AO-associated induction of SIPS needs to be done.

Summarizing, oxidative stress is undoubtedly one of the main causes of premature cellular senescence which can lead to organismal aging. In the case of pro-oxidative conditions, AOs do have protective and therefore anti-aging properties. However, to alleviate the development of oxidative stress it is necessary to use high AO doses which are comparable in the effectiveness with the highly potent endogenous cellular AO enzymes and substances. These high doses can induce antioxidative stress in healthy cells which are maintaining normal redox homeostasis. In this case, AOs do not act as the anti-aging agents anymore and may even cause opposite, pro-aging effects. These observations should be considered, and usage of antioxidants should be better controlled.

## Materials and Methods

### Cell cultures

Human endometrial mesenchymal stem cells (eMSCs) were derived from a desquamated endometrium of menstrual blood from healthy donors^35^. eMSCs possess properties typical for the mesenchymal stem cell cultures: eMSCs are multipotent, express CD13, CD29, CD44, CD73, CD90, CD146, and CD105 surface markers, and are negative for the hematopoietic markers CD34 and CD45^35^. eMSCs were cultivated in DMEM/F12 growth medium containing 10% fetal bovine serum (HyClone), 1% L-glutamine (Gibco) and 1% penicillin-streptomycin (Gibco). eMSCs (from 3 to 15 passages) were maintained in 75 cm^2^ culture flasks at 37 °C in a humidified chamber with 5% CO_2_ and subcultured twice per week. According to our observations^17^, eMSC lines used within the current study become senescent at 20 passage at earliest, so in cells from 3 to 15 passages the effects of replicative senescence have been neglected. In each experiment, the respective control at the same passage as the experimental culture was used in order to address the question of the influence of *in vitro* passaging effects on cell proliferation capacity. For cell-cycle synchronization, cells were accumulated in the G_0_/G_1_ phase of the cell cycle by 24-hour serum deprivation and after that stimulated for proliferation by serum addition.

All experiments were approved by the Ethics Committee of the Almazov National Medical Research Centre (Saint Petersburg, Russia) and performed in accordance with the institutional guidelines. All cell donors signed an informed consent for voluntary participation.

### Cell treatments

Within this study, we used four different synthetic substances with AO capacity: resveratrol and NAC (Sigma-Aldrich), Tempol (Santa Cruz Biotechnologies) and DPI (Calbiochem). The concentrations applied to cells are listed in Table 1. Synchronized cell cultures were treated with AOs either 2 hours (experimental scenario called quiescent cell AO treatment, «Q-AO-treatment») or 14 hours (experimental scenario called proliferating cell AO treatment, «P-AO-treatment») post serum stimulation and then cultivated as it has been previously described.

### Cell viability assay

Cells, harvested with 0.05% trypsin-EDTA solution and suspended in the fresh medium, were stained with 50 *μ*g/ml propidium iodide (Sigma-Aldrich) and analyzed with CytoFLEX flow cytometer (Beckman Coulter, 488 nm laser).

### Cell cycle assay

Cells were harvested with 0.05% trypsin-EDTA solution, suspended in the fresh medium, permeabilized with 0.1% Triton X-100 (Sigma-Aldrich) and stained for 5 min with 2 *μ*g/ml of 4′,6-diamidino-2-phenylindole (DAPI, Sigma-Aldrich). Cell cycle phase distribution was measured with CytoFLEX flow cytometer (Beckman Coulter, 405 nm laser) and analyzed using CytExpert 2.0 software.

### DNA damage assay (flow cytometry)

Cells, harvested with 0.05% trypsin-EDTA solution and suspended in the fresh medium, were washed twice with PBS, fixed and permeabilized using Nuclear Factor Fix and Perm Buffer Set (BioLegend). For specific detection of γH2AX foci accumulation, cells were incubated with anti-γH2AX antibodies (1:200, Abcam) for 1 hour at room temperature in the dark. After washing with PBS, cells were incubated with 1:500 solution of goat-anti-mouse (GAM) Alexa Fluor ® 488 secondary antibodies and 1 *μ*g/ml of DAPI for 30 minutes at room temperature in the dark. The γH2AX foci accumulation, as well as its distribution among the cell cycle phases, were then analyzed with CytoFLEX flow cytometer (Beckman Coulter, 405/488 nm lasers).

### ROS measurements

#### H_2_DCFDA-based assay

Cells were washed with PBS and incubated in the 5 *μ*M staining solution of ROS-sensitive probe 2′,7′-dichlorodihydrofluorescein diacetate (H_2_DCFDA, Invitrogen, D399) in PBS for 30 minutes in the dark at 37 °C. After that, cells were harvested with 0.05% trypsin-EDTA solution, suspended in the fresh medium and immediately analyzed with CytoFLEX flow cytometer (Beckman Coulter, 488 nm laser). Cells were gated by size and granularity on FSC/SSC plot and cell debris was excluded from the analysis.

#### HyPer-based assay

eMSCs were transduced with HyPer-cyto expression vector using commercially available lentiviral particles (Evrogen) and hexadimethrine bromide (Polybrene, Sigma-Aldrich) at a concentration of 5 μg/ml. Transduction was performed 20 hours after cell seeding at a multiplicity of infection (MOI) of 5. Obtained eMSC-HyPer cells stably expressed cyto-HyPer protein and the surface marker set typical for the source eMSC cells^45^. To analyze the impact of AOs from our list (Table 1) on the H_2_O_2_ concentration in cells, eMSC-HyPer cells were incubated with AOs in standard growth conditions (37 °C, 5% CO_2_), then harvested with 0.05% trypsin-EDTA solution, suspended in the previously collected medium containing AOs, and immediately analyzed with flow cytometer (CytoFLEX, Beckman Coulter, 405/488 nm lasers)^45^. Control eMSC-HyPer cells were harvested, then suspended in the previously collected medium and analyzed. Before the analysis, the control cell suspension was split into 3 probes: the first one was analyzed immediately, the second one was analyzed after 5-min incubation with 1 mM of H_2_O_2_, while the rest part of the suspension was incubated for 10 min with 30 mM of dithiothreitol (DTT) and then analyzed. During the analysis, the mean ratio of EX488/FL530 and EX405/FL525 signals (denoted as HyPer-ratio) was measured in gated by size and granularity HyPer + cells. Intracellular peroxide concentration was assessed by calculating HyPer-index, which was quantified in %^42^ as follows:

$$\mathrm{HyPer} \mbox{-} \mathrm{index}=({{\rm{R}}}_{{\rm{cells}}}-{{\rm{R}}}_{{\rm{DTT}}})/({{\rm{R}}}_{{\rm{H2O2}}}-{{\rm{R}}}_{{\rm{DTT}}}),$$

where R_cells_ is a HyPer-ratio measured in cells under investigation, while R_DTT_ and R_H2O2_ are HyPer-ratio values measured in the control cells after incubation with DTT and H_2_O_2_ respectively.

Intracellular pH was controlled in parallel experiments using eMSC cells and the pH-sensitive dye 2′,7′-bis-(2-carboxyethyl)-5-(and-6)-carboxyfluorescein, acetoxymethyl ester (BCECF AM, Invitrogen, B1170) applied in accordance with the manufacturer’s instructions^45^. Cells were analyzed with CytoFLEX flow cytometer (Beckman Coulter, 405/488 nm lasers) by monitoring ratio of EX488/FL530 and EX405/FL525.

### Immunofluorescence assay

Cells were seeded on the coverslips, cultivated for 20 hours, synchronized and treated with AOs as it has been previously described. For the immunofluorescence staining, cells were fixed with 4% formalin in PBS, permeabilized with 0.1% Triton X-100, incubated with 1% bovine serum albumin solution in 0.1% Tween-20 in PBS for 1 hour at room temperature and then treated with following primary antibodies: anti-Ki-67 (1:500), anti-γH2AX (1:500), and anti-phospho-аtaxia telangiectasia mutated kinase (pATM, 1:200, all from Abcam) either for 1 hour at room temperature (for Ki-67) or overnight at 4 °C (for γH2AX and pATM). Then cells were washed with PBS/0.1% Tween-20, incubated with 1:500 solution of secondary antibodies (GAM Alexa Fluor ® 488 for γH2AX and GAM Alexa Fluor ® 568 for pATM and Ki-67, all from Abcam) for 1 hour at room temperature in the dark, washed again with PBS/0.1% Tween-20 and counterstained with 1 μg/mL DAPI. The coverslips were mounted with 2% propylgallate and imaged with either laser-scanning microscope Leica TCS SL or Axiovert 200 M microscope (Carl Zeiss) equipped with a Leica DFC 420 C camera. Image processing was performed using ImageJ software (US National Institutes of Health).

### Western blotting

Cells were lysed as it was described previously^63^. Protein samples were separated by either 4% or 12%-SDS-polyacrylamide gel electrophoresis (SDS-PAGE) with subsequent transfer onto a 0.45 *μ*m nitrocellulose membrane (BioRad). The membrane was stained with Ponceau S Red, cut at the appropriate molecular weights and then blots were blocked with 5% nonfat dry milk in Tris-buffered saline supplemented with 0.05% Tween 20 for 1 hour at room temperature. Blots were then incubated overnight at 4 °C with following primary antibodies: anti-phospho-pRb (Ser807/811, 1:1000), anti-glyceraldehyde 3-phosphate dehydrogenase (GAPDH, 1:1000), anti-phospho-p53 (Ser15, 1:1000), anti-p21Waf1/Cip1 (1:1000), anti-phospho-ATM (Ser1981, 1:1000, all from Cell Signaling), anti- γH2AX (1:1000, Abcam). Blots were washed, incubated with peroxidase-conjugated goat anti-mouse IG (GAM-HRP, 1:10000) and goat anti-rabbit IG (GAR-HRP, 1:10000, both from Cell Signaling) for 1 hour at room temperature and developed with ECL (Thermo Scientific). Hyperfilm (CEA) was from Amersham. For the densitometric analysis of protein bands ImageJ software (US National Institutes of Health) was used.

### SA-β-Gal activity assay

Senescent-associated β-galactosidase (SA-β-Gal) activity was detected in the cells using senescence βgalactosidase staining kit (Cell Signaling Technology) according to manufacturer’s instructions and quantified microscopically by counting X-gal-positive cells in random fields of view with the use of ImageJ software (US National Institutes of Health). No fewer than 500 cells were analyzed for each sample.

### Statistical analysis

All data are presented as the mean values of at least three independent experiments with standard deviations. Statistical significance was calculated using either ANOVA-Tukey test in case of multiple comparisons or Student’s t-test in case of pair comparisons. p-values < 0.05 were considered significant.

## Supplementary information

Supplementary Dataset 1

## Data Availability

Data used in this manuscript will be available to the public.
