# Supplementary material for: High doses of synthetic antioxidants induce premature senescence in cultivated mesenchymal stem cells
Source: Sci Rep. 2019 Feb 4;9:1296. doi: 10.1038/s41598-018-37972-y (PMC6361906; doi:10.1038/s41598-018-37972-y)
Supplement: Supplementary file 1 — Supplementary Dataset 1 [file 41598_2018_37972_MOESM1_ESM.pdf]

High doses of synthetic antioxidants induce premature senescence in cultivated mesenchymal stem cells

Ju.S. Kornienko, I.S. Smirnova, N.A. Pugovkina, Ju.S. Ivanova, M.A. Shilina, T.M. Grinchuk, A.N. Shatrova, N.D. Aksenov, V.V. Zenin, N.N. Nikolsky, and O.G. Lyublinskaya.

Department of Intracellular Signaling and Transport, Institute of Cytology, Russian Academy of Sciences; Tikhoretsky pr. 4, St.Petersburg 194064, Russia

Corresponding author: Olga G. Lyublinskaya, Department of Intracellular Signaling and Transport, Institute of Cytology, Russian Academy of Sciences; Tikhoretsky pr. 4, 194064 St-Petersburg, Russia; o.lyublinskaya@mail.ru; +7-952-226-46-52

Supplementary material

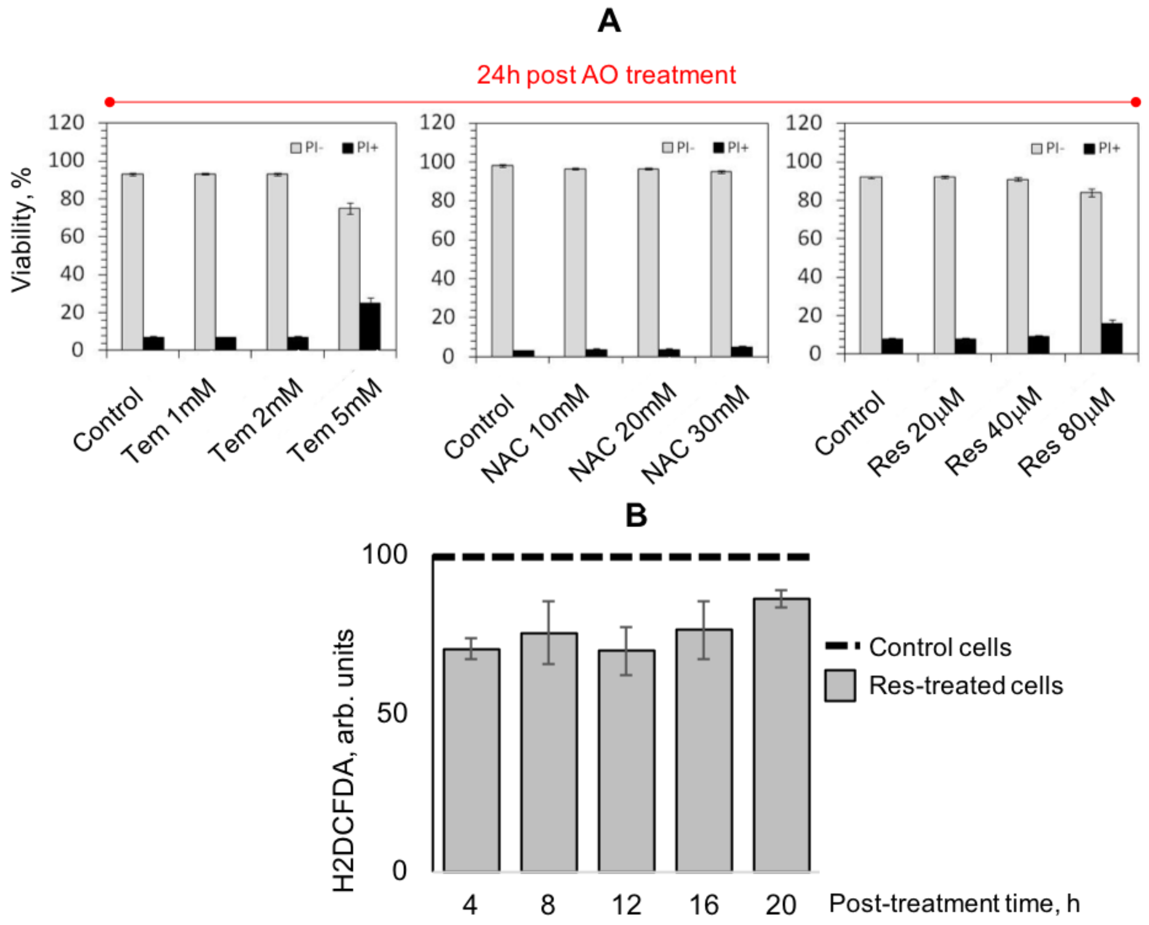

Fig. S-1. Cells treated with antioxidants sustain low ROS level and are not subject to death. (A). The viability of the Q-AO-treated eMSCs after 24-hour incubation with Tempol, NAC and resveratrol (flow cytometry analysis using propidium iodide staining). (B). Flow cytometry analysis of ROS level dynamics in the resveratrol-treated cells incubated with AO within 20 hours and then stained with H_2DCFDA, ROS-sensitive dye. All data are expressed as the mean values ± SD of three independent experiments. Abbreviations: eMSCs, endometrial mesenchymal stem cells; AOs, antioxidants, namely Tempol (Tem, 2 mM), resveratrol (Res, 40 μM), N-acetyl-L-cysteine (NAC, 20mM); Q-AO-treated cells, eMSCs exposed to antioxidants at 2 h post-serum stimulation; PI, propidium iodide; ROS, reactive oxygen species; SD, standard deviation.

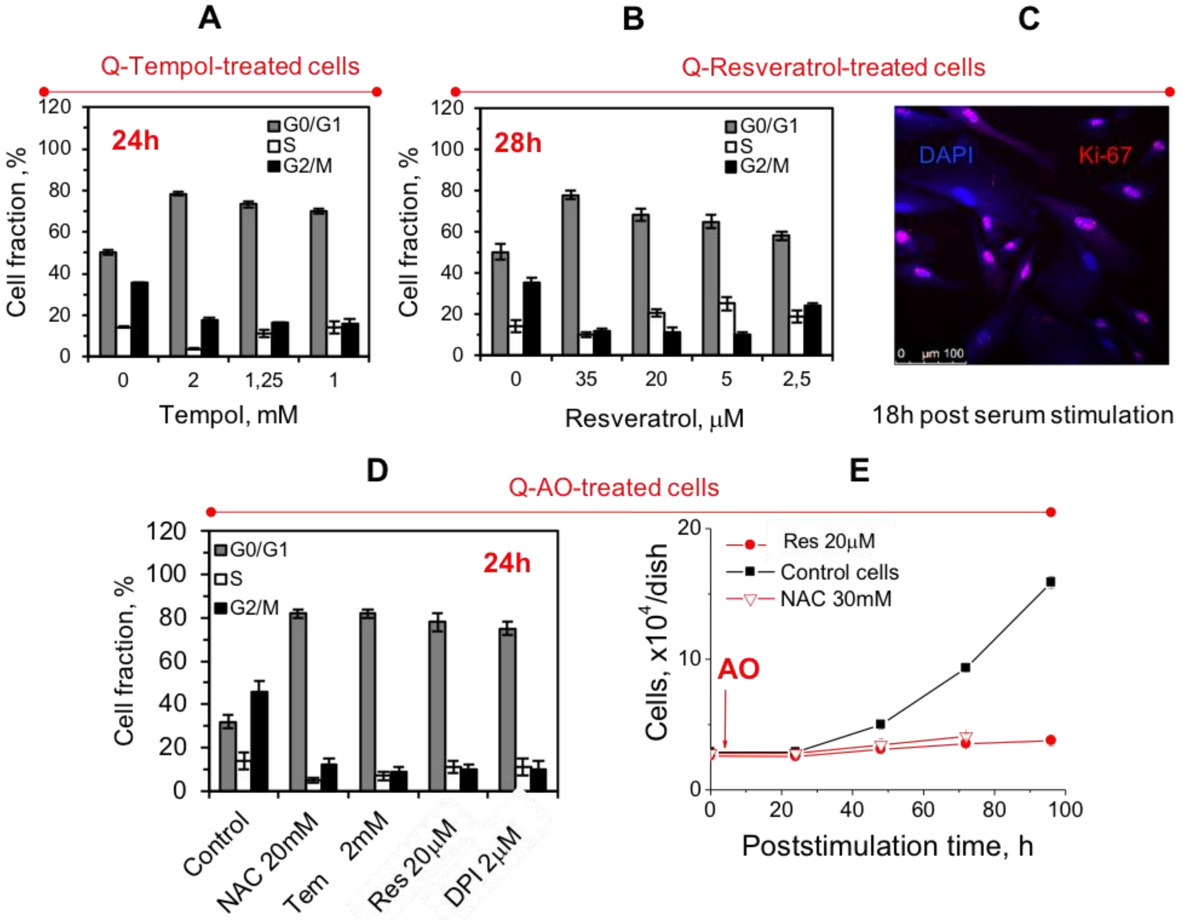

Fig. S-2. Cells treated with antioxidants at quiescence state are arrested in the late G_1 phase. (A, B). Dose-dependent cell cycle blocking after Q-AO-treatment with Tempol (A) and resveratrol (B) (flow cytometry studies). (C). Immunofluorescence analysis of the Ki-67 protein expression in the Q-resveratrol-treated eMSCs (18 h post-serum stimulation). (D). Cell cycle distributions of the control and Q-AO-treated eMSCs after 24-hour treatment: G_1-blocking after Tempol, resveratrol, NAC and DPI applications (flow cytometry studies). (E). Cell growth curves of the control eMSCs and Q-AO-treated cells constantly incubated with resveratrol or NAC. All data are expressed as the mean values ± SD of three independent experiments. Abbreviations: eMSCs, endometrial mesenchymal stem cells; AOs, antioxidants, namely Tempol (Tem, 2 mM), resveratrol (Res, 40 μM), N-acetyl-L-cysteine (NAC, 20mM), Diphenyleneiodonium (DPI, 2μM); Q-AO-treated cells, eMSCs exposed to antioxidants at 2 h post-serum stimulation; DAPI, 4',6-diamidino-2-phenylindole; SD, standard deviation.

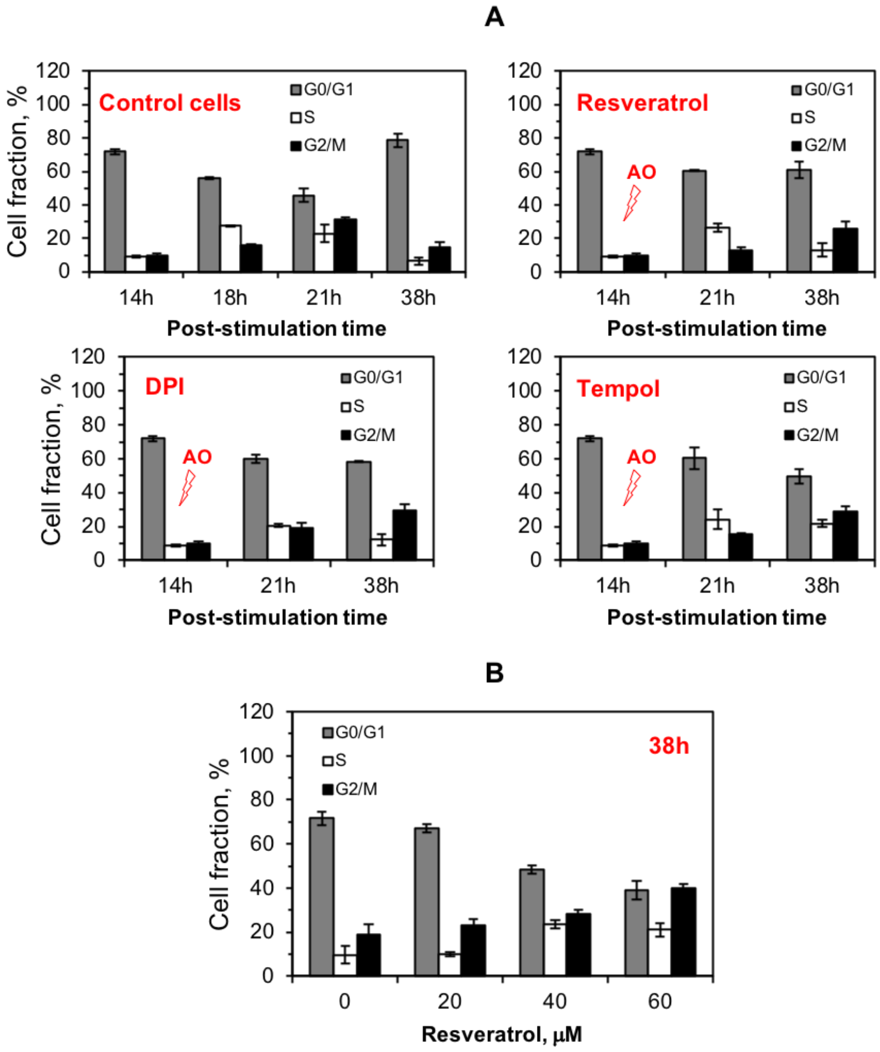

Fig.S-3. Proliferating cells treated with antioxidants progress through the S phase slowly and are eventually accumulated in the G_2/M phase. (A). eMSC cell cycle distributions measured by flow cytometry at different time points after P-AO-treatment with resveratrol (40µM), Diphenyleneiodonium (DPI, 2µM) and Tempol (2mM). (B). Dose-dependent cell cycle blocking after P-AO-treatment with resveratrol (flow cytometry studies). All results are presented as the mean ± SD of three independent experiments. Abbreviations: eMSCs, endometrial mesenchymal stem cells; P-AO-treated cells, eMSCs exposed to antioxidants at 14 h post-serum stimulation; SD, standard deviation.

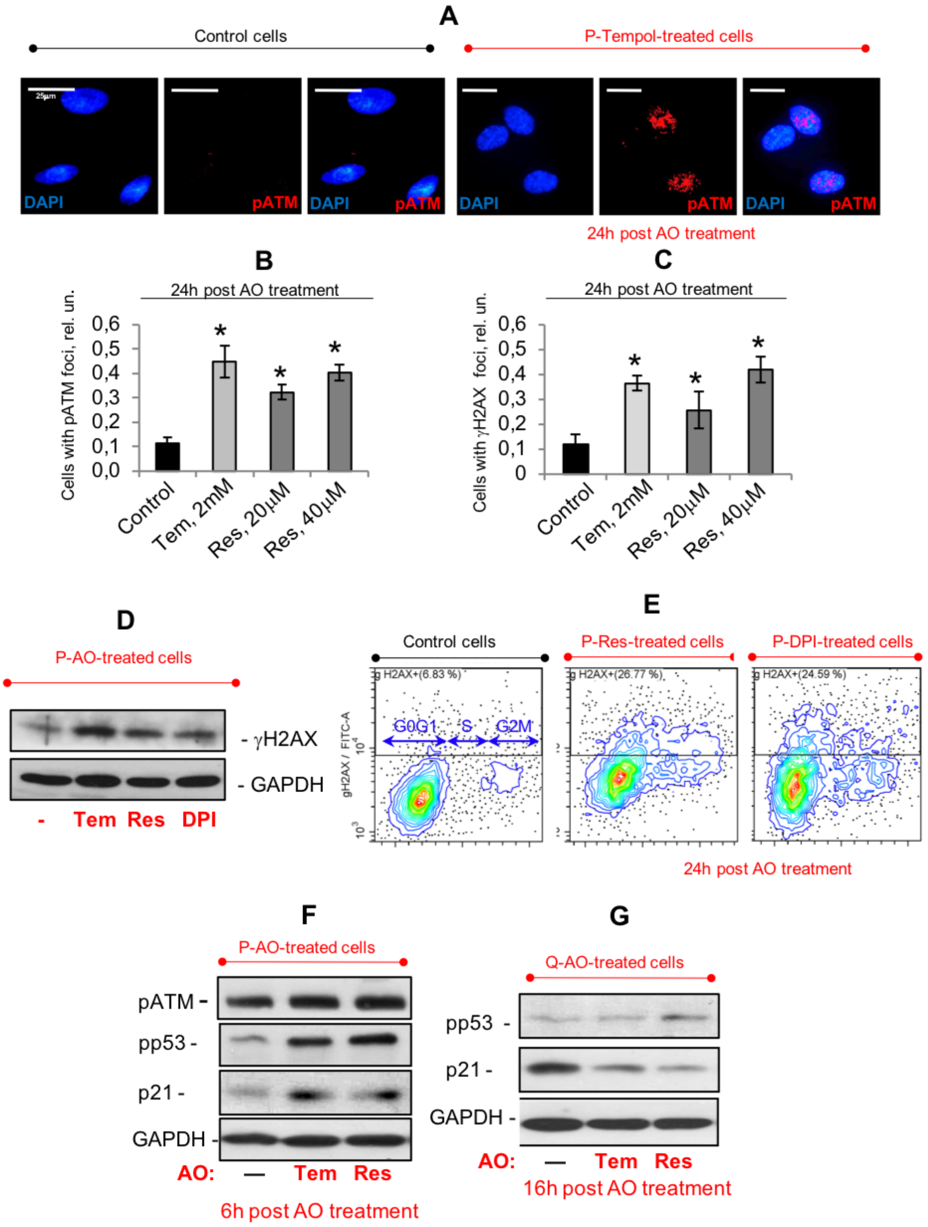

Fig.S-4. Treatments with antioxidants induce DNA damage response activation in P-AO-treated cells. (A). Immunofluorescence analysis of P-AO-treated eMSCs reveals phosphorylation of ATM kinase 24 h after Tempol (2mM) application, scale bar = 25 μm. (B, C). Quantification of pATM (B) and γH2AX (C) foci in P-AO-treated eMSCs after 24-hour incubation with Tempol and resveratrol. In each case, at least 20 images with at least 8 cells were processed. Data are presented as the mean count ± SD. (D) Western blot analysis of γH2AX accumulation in P-AO-treated eMSCs after 24-hour incubation with Tempol (2mM), resveratrol (40μM) or DPI (2μM). (E) Representative dotplots of the cell cycle distributions in the P-AO-treated eMSCs incubated with resveratrol (40μM) and DPI (2μM) for 24 hours and stained for γH2AX. (F, G) Western blot analyses of the DDR-related proteins (pATM, pp53 and p21) expression either in P-AO-treated eMSCs after 6-hour incubation with Tempol (2μM) and resveratrol (40μM) (F), or in Q-AO-treated eMSCs after 16-hour incubation with the same AOs (G). The blot was stained with Ponceau S Red and then cut at the appropriate molecular weights of proteins of interest. * - $p < 0.05$, ANOVA test. Abbreviations: eMSCs, endometrial mesenchymal stem cells; AOs, antioxidants; P-AO-treated cells, eMSCs exposed to AOs at 14 h post-serum stimulation; Q-AO-treated cells, eMSCs exposed to antioxidants at 2 h post-serum stimulation; Tem, Tempol (2mM); Res, resveratrol (40μM); DPI, diphenyleneiodonium (2μM); γH2AX, phosphorylated histone H2AX; p-p53, phosphorylated p53; p21, cyclin-dependent kinase inhibitor $p21^{waf1/cip1}$; p-pRb, phosphorylated retinoblastoma protein; pATM, phosphorylated ATM (ataxia

telangiectasia mutated) kinase; DDR, DNA damage response; DAPI, 4',6-diamidino-2-phenylindole; SD, standard deviation.

.

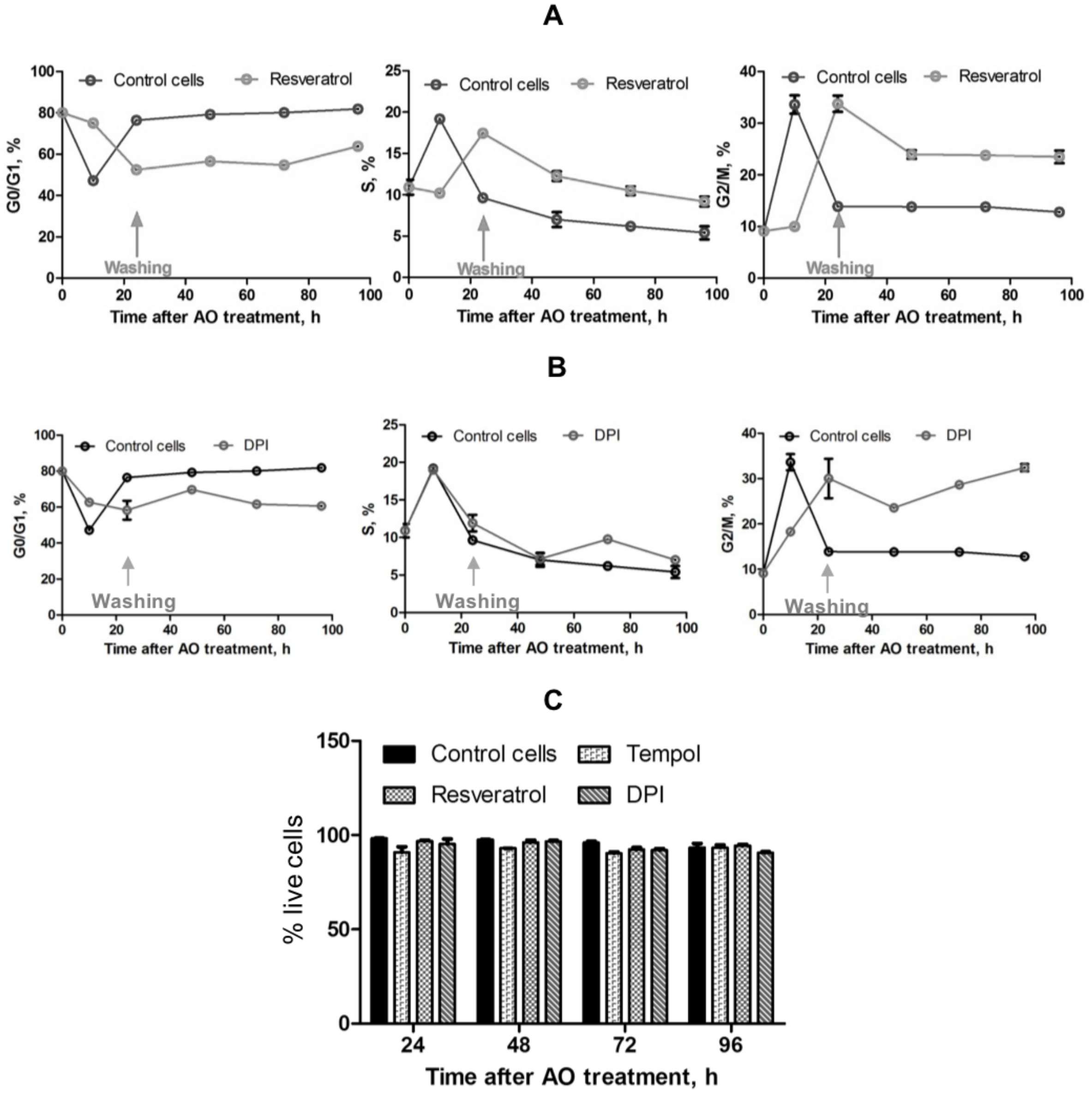

Fig.S-5. P-AO-treated cells irreversibly stop to self-renew without any detectable induction of cell death. (A). Cell cycle progression of the synchronized eMSCs after 24h-P-AO-treatment (with resveratrol) and subsequent washing. Quantification of G_0/G_1, S and G_2/M cell fractions in P-AO-treated eMSCs reveals S-G_2/M-block of cell proliferation. (B). Cell cycle progression of the synchronized eMSCs after 24h-P-AO treatment (with DPI) and subsequent washing. Quantification of G_0/G_1, S, and G_2/M cell fractions in P-AO-treated eMSCs reveals G_2/M-block of cell proliferation. (C). The viability of the P-AO-treated eMSCs after 24h-P-AO-treatment with Tempol, resveratrol and DPI and subsequent washing (flow cytometry analysis using propidium iodide staining). Lack of significant changes in the percentage of live cells was confirmed by ANOVA test. All results are presented as mean ± SD of three independent experiments. Abbreviations: eMSCs, endometrial mesenchymal stem cells; AOs, antioxidants, namely Tempol (Tem, 2 mM), resveratrol (Res, 60 μM), Diphenyleneiodonium (DPI, 2μM); P-AO-treated cells, eMSCs exposed to antioxidants at 14 h post-serum stimulation; SD, standard deviation.
